# Association between oxidative stress and cord serum lipids in relation to delayed cord clamping in term neonates

**DOI:** 10.1186/s12944-017-0599-y

**Published:** 2017-11-09

**Authors:** Asmaa N. Moustafa, Mahmoud H. Ibrahim, Suzan Omar Mousa, Ebtesam E. Hassan, Hashem F. Mohamed, Hend M. Moness

**Affiliations:** 1 Department of Pediatrics, Minia University Hospital, Al-Minya, 61111 Egypt; 2 Department of Obstetrics and Gynecology, Minia University Hospital, Al-Minya, Egypt; 3 Department of Public Health and Preventive Medicine, Minia University Hospital, Al-Minya, Egypt; 4 Department of Clinical Pathology, Minia University Hospital, Al-Minya, Egypt

**Keywords:** Delayed cord clamping, Oxidative stress, Cord lipids, Term neonates

## Abstract

**Background:**

Although delayed cord clamping (DCC) is a recent WHO recommendation, early cord clamping (ECC) is still a routine practice in many countries. Limited researches studied the effect of delayed cord clamping on oxidative stress in term neonates; In this study we aim to assess the influence of cord clamping either early or late on oxidative stress in term neonates and to evaluate the association of oxidative stress and cord blood lipids.

**Methods:**

One-hundred mothers and their term neonates were included in the present study. Umbilical cord blood samples were collected from the umbilical vein and umbilical artery immediately following labor.

**Results:**

Total cholesterol, total triglycerides and phospholipids levels were significantly higher in the ECC group than the DCC group (*p* < 0.001 in all). Plasma total antioxidant status was higher in the DCC group than the ECC group (*p* < 0.001). While, plasma hydroperoxides were lower in the DCC group than the ECC group (*p* < 0.001). Levels of erythrocytes catalase cytosol, superoxide dismutase and glutathione peroxidase were significantly higher in the DCC group than the ECC group (*p* < 0.001).

**Conclusion:**

DCC was associated with a decrease in cord blood lipids and an augmented antioxidant activity. This suggests the protective effect of DCC on the future health of the term neonates and supports the application of DCC in active management of 3rd stage of labor in term neonates.

## Background

During the third stage of labor, the neonate is separated from the placenta by clamping of the umbilical cord. There is a debate concerning the ideal timing for umbilical cord clamping [1]. In the past, the umbilical cord was clamped early in the first 30 s of labor (early cord clamping), as part of active management of the 3rd stage of labor [2]. And this is still widely practiced even in developed countries. Recently, WHO recommended to delay cord clamping one to three minutes after birth [3]. As delayed clamping allows time for a transfer of the fetal blood in the placenta to the neonate at the time of labor. This placental transfusion can provide the neonate with an additional 30% more blood volume and up to 60% more red blood cells [4]. Other benefits of delayed cord clamping include higher hemoglobin concentrations, more iron stores [5], higher red blood cells flow to vital organs, better cardiopulmonary adaptation, and increased duration of early breastfeeding [6].

Labor, though it is a physiological process, carries oxidative stress for both mother and neonate [7]. During pregnancy and labor the oxygen consumption is increased leading to an increase in mitochondrial respiration and loss of electrons produced in the electron transport chain, which leads to accumulation of reactive oxygen species (ROS) [8] With progress of labor, repeated uterine contractions cause uterine ischemia. This is followed by reperfusion, resulting in more production of ROS [9]. Also, Inflammation evoked during labor is a source of pro-inflammatory mediators. These mediators are potent stimulators of ROS production [8].

Adding to the previous factors, the oxidative stress burden is increased on the delivered neonate by the rapid transference from relative hypoxic intrauterine to the extrauterine environment where alveolar pO2 is almost five times higher [10]. Moreover, highly toxic hydroxyl radicals are produced in the delivered neonate due to reduced antioxidant defense [11]. As, the effectiveness of the antioxidant mechanism in the neonate largely depends on the maternal antioxidant system which is highly important in the early minutes of life [12].

Our aim in this study was to assess the influence of cord clamping either early or late on oxidative stress in term neonates and to evaluate the association of oxidative stress and cord blood lipids.

## Method

### Study design and subjects

This analytic observational cross-section study was done in the Obstetrics & Gynecology department and the Pediatric department, Minia University Hospital, Al-Minya, Egypt. The study was conducted over a period of 8 months from the 1st of January till the 30th August 2016. An informed written consent was obtained from parents after the nature and purpose of the study had been explained to them and they were fully understood.

A total of 150 pairs of healthy mothers and their neonates were enrolled in the study and 50 pairs were excluded from the study because 20 of them didn’t meet inclusion criteria, 20 pairs refuse to participate and 10 pairs withdraw before completion of assessment. The remaining 100 pairs were assigned into 2 groups: – Early cord clamping (ECC) group and delayed cord clamping (DCC) group based on computer-generated random numbers. The obstetrician conducting the delivery was informed the group allocation prior to the delivery. In the ECC group, the umbilical cord was clamped within 30 s of fetus expulsion and in the DCC, the umbilical cord was left at 20 cm below the vaginal introitus and then clamped at 2 min after expulsion. The selection of a 2-min interval was based on WHO 2012 recommendations for clamping of the cord [3]. Newborn infants in the DCC were held in their mothers’ arms while waiting for the cord to be clamped.

Mothers included in our study: were disease free, had a normal course of pregnancy, had a BMI of 18–30 at the start of pregnancy, their weight gain was between 8 to 12 kg since pregnancy onset, had a gestational period of 37 to 42 weeks at delivery, had a single fetus in cephalic presentation, and had undergone a spontaneous vaginal delivery. The newborns included were of appropriate weight for gestational age and with Apgar score ≥ 7 at 1st and 5th minutes of life, and normal monitoring results. None of the included mothers experienced any abnormalities during labor and delivered spontaneously. The progress of labor was determined by vaginal examinations every one to two hours and as indicated by clinical conditions. Uterine contractions and fetal heart rate were monitored continuously with cardiotocograph and were normal in all the cases. Clinical parameters were monitored throughout the delivery. Variables such as age, parity, gestational period, and clinical pregnancy outcome were obtained from the mother’s medical history.

### Blood sampling and processing

Arterial and venous umbilical cord samples were drawn. All staff in the delivery unit were trained in the study procedures before the study started. The samples were divided into: 1) About 2.5 ml immediately and quickly were put after cord clamping into vacutainers containing heparin to prevent coagulation (for the assay of the oxidative stress biomarkers). 2) 1.5 ml were put into plain tube for the assay of triglycerides, total cholesterol and phospholipids after centrifugation at 4000×g for 5 min using fully automated clinical chemistry autoanalyzer system Konelab 60i (Thermo Electron Incorporation, Finland).

The vacutainers containing heparin were centrifuged at 1000 _X_ g for 10 min at cooling centrifuge (Beckman, USA), the supernatant was stored at −80 °C for the assay of total antioxidant status **(TAS)** by using kits provided by (Randox Laboratories, UK) and measurement of **Plasma hydro-peroxides** by OxyStat kit ((Biomedica, Austria). The remaining red blood cell pellets were washed with phosphate buffered saline (PBS) (pH 7.4) and diluted to a concentration equivalent to a hematocrit of 40% using PBS, to maintain the number of erythrocytes constant in all the samples, then RBCs were lysed at 4 °C by addition of 5 ml Tris–HCl buffer (pH 7.5) for measurement of **(a) Erythrocyte membrane hydro-peroxides** (this was based on the principle of the rapid peroxide-mediated oxidation of Fe^2+^ to Fe^3+^ which in the presence of xylenol orange, forms a Fe^3 + −^xylenol orange complex measured spectrophotometrically at 560 nm), **(b) measurement of Glutathione peroxidase (GPx) (c) Superoxide dismutase (SOD)** and **(d) Catalase (CAT)** using kits provided by (Randox Laboratories, UK).

Serum bilirubin level was done on the 2nd day after delivery using fully automated clinical chemistry autoanalyzer system Konelab 60i (Thermo Electron Incorporation, Finland).

### Statistical analysis

Data were analyzed using Statistical Package for the Social Sciences (SPSS), Version 19. Quantitative data were presented as a mean and standard deviation, qualitative data presented as a frequency distribution. Chi-square test, Z Test, independent sample t-test and Paired t-test test were used. Pearson correlation was used to test the significant association between lipid profile and oxidative stress, grades of correlation was considered; 0.00: 0.24 (no or weak association), 0.25: 0.49 (mild association), 0.50: 0.74 (moderate association): ≥0.75 (strong association). The probability of less than 0.05 was used as a cut-off point for all significant tests.

## Results

One hundred mother-infant pairs participated in this study over the period from the 1st of January till the 30th August 2016. We compared some clinical characteristics of the participated mothers and newborns in the DCC group versus those in the ECC group, such as maternal age, weight, parity, time of placental separation, presence of postpartum hemorrhage and neonatal weight. These clinical characteristics showed no statistically significant differences between the two studied groups (*p* > 0.05) (Table 1).

**Table 1:** Clinical characteristics of both mothers and newborns in the DCC and ECC groups

Datum	DCC group	ECC group	*p*
	*n* = 50	*n* = 50	
Maternal characteristics			
Maternal Age (years): range	20–35	20–35	0.8
Mean ± SD	26.1 ± 4.4	26 ± 4.6	
Maternal Weight (kg): range	68–90	68–90	0.9
Mean ± SD	77.6 ± 6.8	77.6 ± 7.7	
Parity			
Nulliparous: *n* (%)	17 (34%)	12 (24%)	0.5
Multi-para: *n* (%)	33 (66%)	38 (76%)	
Time of placenta separation (min.): range	3–8	3–8	0.9
Mean ± SD	5.4 ± 1.3	5.4 ± 1.6	
PPH: *n* (%)	4 (8%)	4 (8%)	0.5
Neonatal characteristics			
Neonatal weight (mg): range	2400–4000	2400–3600	0.2
Mean ± SD	3130 ± 464.8	4277.4 ± 5912.5	
Incubated: *n* (%)	0%	0%	–

*DCC* Delayed cord clamping group, *ECC* Early cord clamping group, *PPH* postpartum hemorrhage

*Statistical significance at *p* < 0.05

Regarding to the cord blood serum lipids, total cholesterol, total triglycerides, and phospholipids levels were significantly higher in the ECC group than in the DCC group in both umbilical artery and vein (*p* < 0.001 in all). Moreover, in each of the studied groups, total cholesterol levels were significantly higher in the umbilical venous blood than in the umbilical arterial blood (*p* < 0.001). While only the ECC group showed significantly higher phospholipid levels in the umbilical venous blood than the umbilical arterial blood, (*p* < 0.001) (Table 2).

**Table 2:** Cord blood lipids and serum bilirubin of the studied groups

Variable	DCC group		ECC group		*p*
	*n* = 50		*n* = 50		
	Vein	Artery	Vein	Artery	
Total Cholesterol (mg/dL): mean ± SD	66.1 ± 1.9	60.8 ± 1.1	67.6 ± 6.1	63.1 ± 0.9	0.001*
*p*	0.001*		0.001^*^		
Triglycerides (mg/dL): mean ± SD	41.4 ± 0.9	41.6 ± 1.2	45.4 ± 1.9	45.2 ± 1.9	0.001*
*p*	0.3		0.6		
Phospholipids(mg/dL): mean ± SD	90.4 ± 2.04	90.2 ± 1.8	97.1 ± 1.4	90.6 ± 1.1	0.001*
*p*	0.7		0.001*		
Serum bilirubin at 48 h: mean ± SD	7.2 ± 1.2		7.3 ± 1.04		0.5

*DCC* Delayed cord clamping group, *ECC* Early cord clamping group

*Statistical significance at *p* < 0.05

On comparing the oxidative stress biomarkers in both umbilical venous and arterial plasma, TAS activity levels were significantly higher and plasma hydroperoxides levels were significantly lower in the DCC group than the ECC group (*p* < 0.001 for both). Furthermore, in each of the studied groups, plasma hydroperoxides levels were significantly lower in the umbilical arterial plasma than the umbilical venous plasma (*p* < 0.001 for both) (Table 3).

**Table 3:** Oxidative stress biomarkers of the studied groups

Oxidative stress biomarkers		DCC group	ECC group	p
		*n* = 50	*n* = 50	
		mean ± SD	mean ± SD	
Plasma parameters				
TAS (nmol/mL)	Artery	1.1 ± 0.04	0.9 ± 0.01	0.001*
	Vein	1.2 ± 0.3	0.8 ± 0.01	0.001*
	*p*	0.001*	0.001*	
Plasma hydroperoxides (nmol/mL)	Artery	6.6 ± 0.01	6.9 ± 0.02	0.001*
	Vein	7.7 ± 0.02	8.2 ± 0.02	0.001*
	*p*	0.001*	0.001*	
Erythrocyte parameters				
CAT cytosol (K/seg·mg)	Artery	0.28 ± 0.01	0.23 ± 0.01	0.001*
	Vein	0.32 ± 0.01	0.29 ± 0.01	0.001*
	*p*	0.001*	0.001*	
GPx cytosol (U/mg)	Artery	30.8 ± 0.9	31.7 ± 1.1	0.001*
	Vein	32.1 ± 0.6	33.3 ± .7	0.001*
	*p*	0.001*	0.001*	
SOD cytosol (U/mg)	Artery	243.8 ± 6.7	198.9 ± 4.3	0.001*
	Vein	249.9 ± 8.7	218.9 ± 5.1	0.001*
	*p*	0.001*	0.001*	
Membrane hydroperoxides (nmol/mL)	Artery	22.9 ± 0.8	21.6 ± 0.8	0.001*
	Vein	24.9 ± 0.7	26.4 ± 0.9	0.001*
	*p*	0.001*	0.001*	

*DCC* Delayed cord clamping group, *ECC* Early cord clamping group, *TAS* total antioxidant status, *CAT* catalase, *GPx* glutathione peroxidase, *SOD* superoxide dismutase

*Statistical significance at *p* < 0.05

The levels of erythrocytes CAT cytosol, erythrocytes SOD cytosol, and erythrocytes GPx cytosol were significantly higher in the DCC group than the ECC group (*p* < 0.001 in all); and within each group, their levels were higher in the umbilical veins than in the umbilical arteries (*p* < 0.001 in all) (Table 3).

The levels of erythrocytes membrane hydroperoxides were significantly higher in the umbilical arteries of the DCC group than the umbilical arteries of the ECC group (*p* < 0.001) and were lower in the umbilical veins of DCC group than umbilical veins of ECC group (*p* < 0.001) (Table 3).

Regarding the correlations of cord blood serum triglycerides and oxidative stress biomarkers, triglycerides, in both umbilical arterial and venous blood, showed significant positive associations with plasma hydroperoxides and erythrocytes membrane hydroperoxides (*p* < 0.001 in all). In the meantime, triglycerides showed significant negative associations with plasma TAS and erythrocytes antioxidant enzymes; SOD, CAT and GPx (*p* < 0.001 in all) (Table 4).

**Table 4:** Correlations between cord serum triglycerides and oxidative stress biomarkers

Oxidative stress biomarkers		TG	
		Vein	Artery
TAS			
	*r*	−0.74	−0.75
	*p*	0.001*	0.001*
Plasma hydroperoxides			
	*r*	0.78	0.75
	*p*	0.001*	0.001*
CAT			
	*r*	−0.73	−0.72
	*p*	0.001*	0.001*
GPX			
	*r*	−0.57	−0.41
	*p*	0.001*	0.001*
SOD			
	*r*	−0.74	−0.74
	*p*	0.001*	0.001*
Erythrocytes membrane hydroperoxides			
	*r*	0.50	0.44
	*p*	0.001*	0.001*

*TG* cord serum triglycerides, *TAS* total antioxidant status, *CAT* catalase, *GPx* glutathione peroxidase, *SOD* superoxide dismutase, *r* correlation coefficient

*Statistical significance at *p* < 0.05

## Discussion

Pregnancy and labor exert an oxidative stress on both mother and neonate because of increasing metabolic demands and oxygen requirements. This results in the generation of free radicals as reactive oxygen species (ROS) [13].

ROS attack cellular polyunsaturated membrane lipid, a chain reaction occurs. These reactions continue until scavengers act on these free radicles. These scavengers are called antioxidants [14]. The effectiveness of the antioxidant system in the neonate is dependent on the maternal antioxidant system through the trans-placental transfer of antioxidants to help the neonate to tackle the oxidative stress [15].

Our aim in this study was to assess the influence of cord clamping either early or late on oxidative stress in term neonates and to evaluate the association of oxidative stress and cord blood lipids.

In the present study, DCC did not exert a significant effect on the maternal outcomes, such as postpartum hemorrhage. This was in accordance with Anderson et al. in 2013 [16]. This confirms that delayed cord clamping is a safe maneuver that does not increase the risk of maternal blood loss after a normal labor.

On assessing the effect of DCC on the neonatal outcome, we found that there were no statistically significant differences between the DCC group and the ECC group regarding the need for admission to NICU or the newborns 2nd day bilirubin level. This was in agreement with previous studies [17, 18]. So, DCC does not increase the risk of NICU admission [17] or hyperbilirubinemia [18].

In this study, total cholesterol levels were higher in the umbilical veins than in the umbilical arteries in both groups. And the phospholipids in the ECC group were also higher in the umbilical veins than in the arteries. This means that the mother transfers more lipids to the fetus than the fetus transfer to the mother. A study by Diaz-Castro et al. in 2015 was consistent with our results [19]. This may be attributed to physiological hyperlipidemia occurring in healthy pregnant women especially in the second half of pregnancy [20]. Also, Diaz-Castro et al., explained the high cord lipid by the high necessity of lipids for fetal growth [19]. However, other maternal and intrauterine factors might directly impact neonatal lipid parameters [21].

Cord blood lipids were lower in the DCC group than the ECC group. Which means that DCC decrease lipid transfer from mother to fetus. This was in agreement with us Florido et al. in 2015 [22], and may be attributed to the minimizing effect of DCC on oxidative stress.

We found plasma hydroperoxides and erythrocytes membrane hydroperoxides concentrations to be higher in umbilical veins than arteries in both groups reflecting that the neonates are affected by oxidative stress damage exerted upon them from the process of labor itself and from the mother much more than oxidative stress in the neonates themselves. Similar results were reported by Arguelles et al., [23]. Peroxidases increase in oxidative stress as a result of increased ROS generation. These ROS interact with polyunsaturated fatty acids in membranes or lipoprotein and start the process of lipid peroxidation and production of chemicals like peroxidases.

In this study, the values of erythrocytes SOD, CAT, and GPx enzymes were higher in the umbilical veins than the arteries in both groups. Their lower levels in the neonates may be due to their consumption in neutralization of free radicals produced during the labor. As, they represent part of the antioxidant system generated by oxidative stress [24]. Moreover, their levels were higher in the DCC group than the EEC group. This may affirm the beneficial effects of DCC maneuver, as it augments the antioxidant enzyme system in the neonate.

In the ECC group, TAS level was lower in umbilical venous blood than arterial blood, which can be explained by its consumption in the neutralization of the high plasma peroxides content present in the ECC group. On the contrary, in the DCC group, the level of TAS was higher in the umbilical venous blood than the arterial blood, which supports the protective effect of DCC.

In this study, cord blood serum triglycerides had significantly negative correlations with TAS, SOD, CAT and GPx levels and significantly positive correlations with plasma hydroperoxides and erythrocytes membrane hydroperoxides. This represents the complex interactions and the multi-systemic responses of the oxidative stress-hyperlipidemia relationship. As, hyperlipidemia was found to enhance lipid peroxidation and to decrease antioxidant enzyme activities, which in turn increase the oxidative stress damage [25].

## Conclusion

DCC decreases oxidative stress damage in term neonates exposed to during labor. As, DCC augments the antioxidant system by enhancing the activities of antioxidant enzymes SOD, CAT, GPx and TAS, on one hand and decreasing hyperlipidemia, on the other hand. These findings support the beneficial effects of DCC on the future health of term neonates. So, we support the WHO recommendation of DCC in the active management of 3rd stage of labor.

## Abbreviations

CAT: Catalase
DCC: Delayed cord clamping
ECC: Early cord clamping
GPx: Erythrocytes glutathione peroxidase
ROS: Reactive oxygen species
SOD: Erythrocytes superoxide dismutase
TAS: Total antioxidant status
